# Nutritional Status of Patients on Maintenance Hemodialysis at Muhimbili National Hospital in Dar es Salaam, Tanzania: A Cross-Sectional Study

**DOI:** 10.1155/2021/6672185

**Published:** 2021-05-22

**Authors:** Puneet Bramania, Paschal Ruggajo, Rimal Bramania, Muhiddin Mahmoud, Francis Furia

**Affiliations:** ^1^Muhimbili University of Health and Allied Sciences, Dar es Salaam, Tanzania; ^2^Renal Unit, Muhimbili National Hospital, Dar es Salaam, Tanzania

## Abstract

**Background:**

Patients on hemodialysis therapy are at high risk of malnutrition which is attributed to multiple factors. Protein-energy malnutrition in these patients confers poor clinical outcomes. This study investigated the nutritional status of patients on maintenance hemodialysis at Muhimbili National Hospital in Dar es Salaam, Tanzania.

**Methods:**

A cross-sectional descriptive study was done among 160 adult patients on maintenance hemodialysis therapy. Data concerning patients' personal, medical, dietary, and hemodialysis-related information were collected. Patients' anthropometric and laboratory tests (complete blood count, albumin, total cholesterol, creatinine, and urea) were measured. The quantitative Subjective Global Assessment (SGA) dialysis malnutrition score (DMS) was used to assess their nutritional status. Data analysis was done using the SPSS software version 20.

**Results:**

Among the 160 hemodialysis patients, 49 (30.6%) were female. Patients' mean age was 52.2 ± 13.3 years. The median duration on hemodialysis was 18 (8.25–29.75) months. Malnutrition was present in 98 (61.2%) of the patients. Severe malnutrition was found in only 3 (1.9%) patients and 16.9% were underweight. The longer duration on hemodialysis, having diabetes mellitus, and being single were associated with increased risk for malnutrition in multivariate logistic regression. Malnourished patients had significantly lower dry weight, body mass index, mid-upper arm circumference, waist circumference, albumin, total cholesterol, and creatinine levels.

**Conclusion:**

Malnutrition is very common among hemodialysis patients at Muhimbili National Hospital, especially those on longer duration of hemodialysis, and diabetic patients. We recommend that hemodialysis patients should be regularly assessed for malnutrition and appropriately treated which if left unattended heralds worse outcomes.

## 1. Introduction

The burden of Chronic Kidney Disease (CKD) in Tanzania is high [1, 2]. The number of patients receiving hemodialysis (HD) therapy in Tanzania has increased in recent years [3]. Hemodialysis services are expensive, National Health Insurance Fund (NHIF) provides reimbursement for most patients in Tanzania, and patients who are not subscribers of NHIF face challenges in meeting the costs and usually receive inadequate HD therapy [3, 4]. The persistence of uremic symptoms including nausea, vomiting, and anorexia is common among patients receiving inadequate dialysis and affects dietary intake significantly. Moreover, HD therapy is accompanied by inflammatory and catabolic processes, which result in protein-energy wasting (PEW) [5, 6].

Malnutrition affects about 40% of CKD patients with early manifestation in CKD stage three particularly with an estimated glomerular filtration rate below 35 ml/min [7]. Hemodialysis patients have a high burden of malnutrition (20–61.8%) [8, 9]; a previous study done in Dar es Salaam, Tanzania, reported underweight in 9.8% of the patients on maintenance HD [10]. Mortality in HD patients has been associated with body size as well as the duration of dialysis and Kt/V [11]. Stenvinkel et al. reported a paradoxical effect of BMI on mortality; patients with lower BMI had higher odds of mortality [12].

Two types of malnutrition have been described among ESRD patients: type 1 in which inadequate intake from anorexia, nausea, and vomiting leads to under-nutrition; it generally improves with adequate dialysis and appropriate nutritional support; type 2 is thought to be mediated by chronic inflammation and may not always improve with adequate dietary intake or dialysis [13, 14]. Although dietary restrictions are an integral part of the management of HD patients, they may deprive them of essential nutrients [15, 16]. Malnutrition is therefore an expected complication in patients receiving maintenance HD therapy.

Due to multiple limitations and differences in eligibility for renal transplants in our setting, this therapy is not possible for most patients. They require maintenance HD for the rest of their lives; thenceforth they are prone to malnutrition. Prompt assessment and treatment of malnutrition can reduce the associated morbidities in these patients. We aimed to determine the prevalence of malnutrition and its associated risk factors among patients on maintenance hemodialysis at Muhimbili National Hospital (MNH) in Dar es Salaam, Tanzania.

## 2. Materials and Methods

### 2.1. Study Design and Duration

This was a cross-sectional descriptive study. Data were collected for 3 months from September to November 2019.

### 2.2. Study Site and Study Population

The study was done at two hemodialysis centers of MNH located at Upanga and Mloganzila in Dar es Salaam city in Tanzania. MNH is the national referral and teaching hospital in Tanzania.

The study included all adult patients (18 years or above) on maintenance hemodialysis (that is, for at least 3 months). Patients with mental disorders and altered levels of consciousness, completely bedbound, or with severe respiratory distress were excluded.

### 2.3. Sample Size and Sampling Method

The sample size was computed using Kish and Leslie formula, with a prevalence of malnutrition of 85.2% [based on quantitative Subjective Global Assessment (SGA)] among hemodialysis patients in Egypt [17]. At a level of significance, alpha (*α*) = 0.05, a margin of error of 5.5%, the minimum calculated sample size was 160 patients. Simple random sampling was done using Stat Trek's random number generator after assigning numbers to all patients [18].

### 2.4. Data Collection Methods

Data were collected using a semi-structured questionnaire, which included questions about patients' demographic, clinical/medical history, and hemodialysis information. The quantitative SGA dialysis malnutrition score (DMS) was used to evaluate the nutritional status of patients. This was adopted from the invented SGA as described by Kalantar et al. [19]. It consists of seven aspects: dietary intake, gastrointestinal symptoms, weight change, comorbidities, functional capacity, subcutaneous fat, and signs of muscle wasting. Each of these parameters was given a score ranging from 0 (normal) to 5 (severe). The sum of individual scores of these parameters represented the DMS. A total score of 7 was considered normal and 35 corresponded to severest malnutrition. Patients' post-dialysis dry weight was measured using a standard weighing scale. A stadiometer was used to determine the height to the nearest centimeter. A tape measure was used to measure the mid-upper arm circumference (MUAC) and waist circumference to the nearest millimeter. The body mass index (BMI) was computed using the dry weight (kg) and height (m) and was expressed in kg/m^2^: BMI = dry weight (kg)/[height (m)]^2^

Patients' dry weight three months ago was searched from their hemodialysis records. The percentage change in weight as compared to the current dry weight was then calculated. The degree of muscle wasting was assessed at the temple, clavicle, ribs, scapula, quadriceps, knee, and interosseous. The degree of subcutaneous fat loss was assessed underneath the eyes, at the biceps and triceps, and on the chest. The frequencies of various food intake and commonly avoided foods were also enquired.

### 2.5. Laboratory Tests

Laboratory measures include complete blood count (CBC), serum albumin, total cholesterol, pre- and post-dialysis urea, and creatinine. These were tested at the MNH Central laboratory. The machines used to analyze the blood samples were CELL DYN 3700 for CBC and ARCHITECT PLUS for serum biochemistry.

### 2.6. Study Variables

The main outcome variable was malnutrition as defined by an aggregate DMS of 11 or more. A score ranging from 7 to 10 was considered as normal to mild malnutrition, a score of 11–22 was considered as moderate *malnutrition*, and DMS of 23–35 was considered as severe malnutrition. These grades with respective scores were replicated from the study done in Egypt [17]. The nutritional status as assessed by the body size (using BMI) was categorized as underweight (BMI<18.5 kg/m^2^), well-nourished (BMI = 18.5 to 24.9 kg/m^2^), and overweight/obese (BMI of 25 kg/m^2^ or above). The comorbidities (hypertension, diabetes mellitus, hepatitis b, and human immunodeficiency virus (HIV) infection) were defined as being diagnosed to have the illness, use of respective medications, and or from patient's medical records. The “urea reduction ratio (URR)” was used to describe the dialysis adequacy. It was computed using the formula: URR = (pre-dialysis urea − post-dialysis urea)/pre-dialysis urea × 100%. Inadequate dialysis was defined as a URR of below 65%.

### 2.7. Data Management and Analysis

Data entry, cleaning, and analysis were carried out using Statistical Package of Social Sciences (SPSS) version 20. Categorical and continuous variables were compared using Chi-square and Student's *t*-test, respectively, and logistic regression analysis (univariate and multivariate) was used to determine factors associated with malnutrition. Only variables with a univariate *p* value below 0.2 were included in the multivariate analysis model. A *p* value of less than 0.05 was considered statistically significant.

### 2.8. Ethical Considerations

Muhimbili University of Health and Allied Sciences (MUHAS) Institutional Review Board and Muhimbili National Hospital's administration gave ethical approval and permission, respectively, for this study. All participants provided written informed consent prior to recruitment.

## 3. Results

### 3.1. Sociodemographic and Clinical Characteristics of Study Subjects

Among the 160 patients on maintenance HD, 49 (30.6%) were female. Almost one-third (34.3%) were aged above 60 years, and the mean age (±SD) of patients was 52.2 (±13.3) years. The median duration on HD was 18 (8.25–29.75) months. Most of them were living with a partner (85%), and health insurance was the main mode of payment for dialysis services. Hypertension was present in 96.2% of patients, whereas 43.1% had diabetes mellitus and 9.4% had HIV infection (Table 1).

### 3.2. Nutritional Status of Hemodialysis Patients

**Figure 1:**
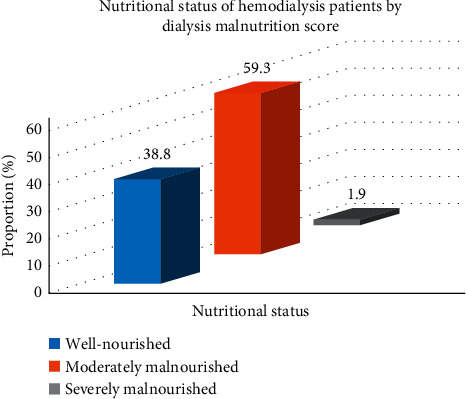
Nutritional status of hemodialysis patients by dialysis malnutrition score.

The dialysis malnutrition score (DMS) ranged from 7 to 30. The mean DMS was 12.3 ± 3.6. Malnutrition (defined by DMS ≥ 11) was prevalent in 98 (61.2%) and was categorized as moderate in 59.3% and severe in 1.9% of the patients (Figure 1).

**Figure 2:**
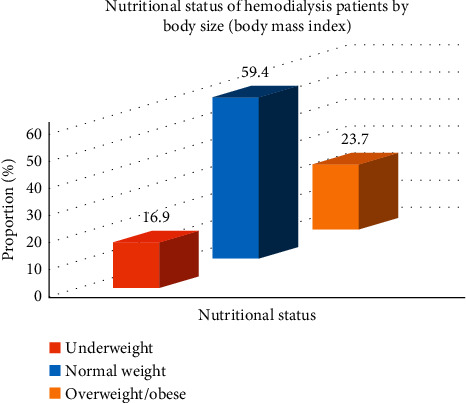
Nutritional status of hemodialysis patients by body size (body mass index).

Body size as assessed by BMI showed that 16.9% of the patients were underweight (Figure 2).

Loss of dry-weight as compared to three months prior to the study was present in 77.5% of the patients. Patients with severe (more than 5%) dry-weight loss had lower mean serum albumin as opposed to those without any dry-weight loss (3.33 g/dl versus 3.75 g/dl, *p* < 0.05).

Malnourished patients had significantly lower BMI, MUAC, and waist circumference. The biochemical markers reflecting nutritional status (albumin, creatinine, and total cholesterol) were all lower among malnourished patients. Hemoglobin, urea, and URR did not differ significantly between well-nourished and malnourished patients (Table 2).

### 3.3. Factors Associated with Malnutrition among Hemodialysis Patients

In univariate logistic regression analysis, only the duration of hemodialysis was significantly associated with malnutrition. (Table 1). Variables with a univariate *p* value < 0.2 were included in multivariate analysis. This revealed that longer duration on HD, diabetic patients, and those who were single (not living with a partner) associated with malnutrition. Compared to those on HD for less than 1 year, patients on HD for more than 4 years had 30 times the odds of having malnutrition. Similarly, diabetic patients and those who were single had significantly higher odds of malnutrition (Table 3).

### 3.4. Dietary Intake of Hemodialysis Patients

Eighty six (53.8%) participants reported no change in dietary intake whereas 74 (46.2%) participants reported sub-optimal dietary intake of varying degree. Almost one-third (31.2%) had some gastrointestinal symptoms (nausea, vomiting, and anorexia) that limited their dietary intake. More than three-fourths of the patients reported at least one consumption per week of protein rich foods (eggs, fish, chicken), carbohydrates (bread, buns, porridge), fried foods, and some green vegetables. Fruits reported to be eaten commonly included apples (91.2%), cucumbers (87.5%), and watermelons (88%). The commonly avoided foods included meat (91.3%), bananas (86.3%), yoghurt (76.3%), potatoes (62.5%), oranges (89.4%), pawpaw (88.8%), and mangoes (93.1%). No quantification of individual meal food items was carried out.

## 4. Discussion

Malnutrition is common among patients receiving hemodialysis therapy and is usually accompanied by high morbidity and mortality [5, 6, 11–13]. In this study, we evaluated 160 adult patients on maintenance hemodialysis. Malnutrition was prevalent in 61.2% (moderate in 59.3%, severe in 1.9%); this is consistent with findings from other studies [17, 20, 21]. Long duration of hemodialysis, having diabetes mellitus, and being single were independently associated with malnutrition.

The prevalence of malnutrition in our study is lower than that reported in a similar study (85.2%) among HD patients in Egypt [17]; this difference might be attributed to the longer duration of HD in Egyptian study participants, in which more than a third (34.7%) of patients had been on HD therapy for at least four years compared to 8.8% in our study.

In our study, patients on dialysis for longer than 4 years were more likely to have malnutrition compared to those on dialysis for less than 1 year. Dialysis duration has been found to correlate with modified quantitative SGA score in a study done in Turkey among geriatric hemodialysis patients [20]. Long-term dialysis therapy can intensify the chronic inflammation-mediated proteolysis, hyper-metabolism, and nutrient losses that eventually contribute to malnutrition [5, 6, 13, 14, 17]. Some of these processes are unavoidable; thus, malnutrition may occur despite adequate dietary intake, making timely renal transplantation when possible to be the most effective intervention to minimize the metabolic complications.

Adequacy of HD reduces the magnitude of protein-energy malnutrition by decreasing the exposure to uremic toxins and cytokines that are implicated in anorexia, vomiting, and inflammation [5, 6, 11]. Most patients in our study were adequately dialyzed (76.2%) and a large proportion was on the standard three times per week HD therapy. The extent of inadequate HD in our study was lower when compared to a previous study done in Tanzania [10]. Besides inadequate dialysis, other factors that induce PEW include metabolic acidosis, hormonal imbalances (insulin resistance, increased glucocorticoid activity), increased energy expenditure, depression, and co-morbidities [5, 6, 13, 14].

Our study reveals significant associations between nutritional status (as assessed by SGA dialysis malnutrition score) and BMI, MUAC, and albumin. This is consistent with findings from the study done in Turkish geriatric hemodialysis patients in which modified SGA score linearly correlated with BMI, albumin, and markers of muscle mass such as mid-arm circumference and mid-arm muscle circumference [20].

Body mass index which is easily measured can be used to determine the nutrition status of patients on HD therapy; Agboton et al. in their study conducted among HD patients in Benin reported a significant correlation between BMI and SGA [21]. We similarly found lower mean BMI amongst patients who were malnourished based on the DMS. In addition, malnourished patients had lower MUAC. This is consistent with a study by Moussa et al. in Niger [22]. These findings reflect the muscle and fat loss associated with malnutrition. Malnourished patients had lower serum albumin (an indicator of visceral protein), creatinine (a marker of muscle mass), and total cholesterol reflecting fat stores. The mean serum albumin of HD patients in our study (3.72 ± 0.48 g/dl) was lower than the recommended target of 4 g/dl by the National Kidney Foundation Kidney Disease Outcomes Quality Initiative [23].

Almost half of the patients reported to have varying degrees of sub-optimal dietary intake. Dietary restrictions and poor adherence to recommended dietary plans contribute to malnutrition among HD patients [16, 17, 24]. Most dietary recommendations in literature are derived from developed counties and may not be applicable for patients in resource-limited settings particularly in sub-Saharan Africa; therefore it is important to develop locally appropriate diet recommendations to ensure adequate nutrition.

In our study, patients who were single had a higher likelihood of being malnourished; this may reflect a poor social support among these patients as opposed to those who were living with their spouses. Patients with malnutrition have been reported to have poor quality of life [25]. In our study, diabetic patients had high odds of malnutrition. Restrictions of carbohydrate-rich foods in these patients can limit optimal calorie intake. Ensuring an adequate calorie and protein intake is an essential component in the treatment of patients on chronic HD therapy [23, 24].

Maintaining the optimum nutrition of HD patients seems very difficult due to the multifactorial causation of malnutrition. They need proper assessment and an individualized approach. This includes oral or intradialytic nutrient supplementation, appetite stimulants (e.g., anabolic steroids), maintaining adequate HD, anti-inflammatory therapies (statins, eicosanoids, *α*-linolenic), and anti-oxidant drugs (like vitamin E, and pentoxifylline) [14, 15]. This needs awareness among healthcare providers, dieticians, and patients themselves.

This is the first study in the country to investigate the nutritional status of patients on maintenance HD and its related factors using the dialysis malnutrition scoring. It has some limitations: including limited generalizability of the findings because of the single center study setting, the three-month retrospective records of dry weight of participants, the accuracy of these records may be ascertained and the duration of dry weight record is not in line with recommended six months as per DMS. Lack of meal and food content quantification in this study has limited inference regarding adequacy of dietary intake of participants.

## 5. Conclusion

Malnutrition is very common among hemodialysis patients at Muhimbili National Hospital especially among those on longer duration of hemodialysis and diabetic patients. Most patients had moderate malnutrition, and severe malnutrition was infrequent. Malnourished patients had lower albumin, creatinine, and total cholesterol levels. We recommend that hemodialysis patients should be screened regularly for malnutrition and should be timely and appropriately treated which if left unattended it can lead to worse outcomes.

## Figures and Tables

**Table 1:** Sample distribution, prevalence, and unadjusted odds of malnutrition in relation to the sociodemographic, hemodialysis-related, and clinical variables. *N* = 160.

Characteristics	Sample distribution *n*(*p*%)	Proportion malnourished *n*(*p*%)	Crude odds ratio (COR) (95% CI)	*p* value
Sociodemographic characteristics				
*Age (years)* 18–39 40–59 ≥60	30 (18.8%) 75 (46.9%) 55 (34.3%)	21/30 (70%) 42/75 (56%) 35/55 (63.6%)	1 0.55 (0.22–1.35) 0.75 (0.29–1.95)	0.19 0.56
*Gender* Male Female	111 (69.4%) 49 (30.6%)	65/111 (58.6%) 33/49 (67.3%)	1 1.46 (0.72–2.96)	0.29
*Marital status* Living with a partner Single	136 (85.0%) 24 (15.0%)	79/136 (58.1%) 19/24 (79.2%)	1 2.74 (0.97–7.78)	0.06
*Educational level* Higher Secondary None or primary	62 (38.8%) 57 (35.6%) 41 (25.6%)	36/62 (58.1%) 33/57 (57.9%) 29/41 (70.7%)	1 0.99 (0.48–2.06) 1.75 (0.75–4.05)	0.99 0.19
*Employment status* Currently working Retired/stopped Not employed	24 (15.0%) 118 (73.8%) 18 (11.2%)	14/24 (58.3%) 70/118 (59.3%) 14/18 (77.8%)	1 1.04 (0.43–2.54) 2.5 (0.63–9.9)	0.33
*Payment type* Health insurance Cash payment	144 (90.0%) 16 (10.0%)	87/144 (60.4%) 11/16 (68.8%)	1 1.44 (0.48–4.37)	0.52

Hemodialysis-related factors and medical conditions				
*Duration on HD* <1 year 1–4 years >4 years	54 (33.7%) 92 (57.5%) 14 (8.8%)	22/54 (40.7%) 63/92 (68.5%) 13/14 (92.9%)	1 3.16 (1.57–6.36) 18.9 (2.3–155.2)	0.001
*Frequency of HD* Thrice/week Twice/week	132 (82.5%)^a^28 (17.5%)	78/132 (59.1%) 20/28 (71.4%)	1 1.73 (0.71–4.22)	0.23
*Vascular access* AV fistula Dialysis catheter	76 (47.5%) 84 (52.5%)	48/76 (63.2%) 50/84 (59.5%)	1 0.86 (0.45–1.62)	0.64
*Dialysis adequacy* Adequate Inadequate	122 (76.2%) 38 (23.8%)	74/122 (60.7%) 24/38 (63.2%)	1 1.11 (0.52–2.36)	0.78
*Diabetes mellitus* Present Absent	69 (43.1%) 91 (56.9%)	47/69 (68.1%) 51/91 (56.0%)	1.68 (0.87–3.22) 1	0.12
*Hypertension* Present Absent	154 (96.2%) 6 (3.8%)	94/154 (61.0%) 4/6 (66.7%)	0.78 (0.14–4.41) 1	0.78
*Hepatitis B infection* Present Absent	9 (5.6%) 151 (94.4%)	6/9 (66.7%) 92/151 (60.9%)	1.28 (0.31–5.33) 1	0.73
*HIV infection* Present Absent	15 (9.4%) 145 (90.6%)	12/15 (80.0%) 86/145 (59.3%)	2.74 (0.74–10.1) 1	0.13

COR: crude/unadjusted odds ratio; CI: confidence interval.

**Table 2:** Comparison of anthropometric and biochemical parameters with nutritional status of hemodialysis patients. *N* = 160.

Parameters	Total *N* = 160	Well-nourished *n* = 62 (38.8%)	Malnourished *n* = 98 (61.2%)	*p* value
Dry weight (kg)	63.2 ± 11.9	69.7 ± 11.5	59.1 ± 10.3	<0.001
BMI (kg/m^2^)	22.5 ± 4.0	24.8 ± 4.1	21.0 ± 3.1	<0.001
MUAC (cm)	25.0 ± 3.9	27.3 ± 3.5	23.5 ± 3.4	<0.001
Waist circumference (cm)	85.8 ± 14.2	91.9 ± 14.7	81.8 ± 12.4	<0.001
Serum albumin (g/dl)	3.72 ± 0.48	3.84 ± 0.52	3.65 ± 0.45	0.015
Total cholesterol (mg/dl)	151.9 ± 39.7	162.5 ± 40.1	145.2 ± 38.2	0.007
Hemoglobin (g/dl)	9.3 ± 1.9	9.7 ± 1.9	9.1 ± 1.9	0.058
Pre-HD creatinine (*µ*mol/l)	845 ± 377	895 ± 436	814 ± 332	0.183
Post-HD creatinine (*µ*mol/l)	317 ± 177	355 ± 218	293 ± 142	0.032
Pre-HD urea (mmol/l)	19.4 ± 11.3	20.1 ± 11.9	18.9 ± 10.9	0.504
Post-HD urea (mmol/l)	5.9 ± 4.3	6.2 ± 5.0	5.6 ± 3.8	0.374
Urea reduction rate (%)	70.2 ± 11.3	70.2 ± 11.5	70.2 ± 11.3	0.985

Test-statistic: Student's *t*-test.

**Table 3:** Multivariate logistic regression analysis to show associated risk factors for malnutrition among hemodialysis patients (*N* = 160).

Characteristics	Adjusted odds ratio (AOR) (95% CI)	*p* value
*Age (years)* 18–39 40–59 ≥60	1 0.72 (0.22–2.33) 0.86 (0.25–2.90)	0.58 0.80

*Marital status* Living with a partner Single	1 4.33 (1.23–15.3)	0.02

*Educational level* Higher Secondary None or primary	1 1.43 (0.62–3.32) 2.05 (0.79–5.32)	0.41 0.14

*Duration of HD* <1 year 1–4 years ≥4 years	1 3.64 (1.70–7.82) 30.0 (3.35–270)	0.001 0.002

*Diabetes mellitus* Present Absent	2.39 (1.09–5.22) 1	0.03

*HIV infection* Present Absent	3.72 (0.90–15.32) 1	0.07

## Data Availability

The datasets are not publicly available. However, they are available from the corresponding author on reasonable request.
